# Dysregulated immunologic landscape of the early host response in melioidosis

**DOI:** 10.1172/jci.insight.179106

**Published:** 2024-08-20

**Authors:** Patpong Rongkard, Lu Xia, Barbara Kronsteiner, Thatcha Yimthin, Rungnapa Phunpang, Adul Dulsuk, Viriya Hantrakun, Gumphol Wongsuvan, Parinya Chamnan, Lara Lovelace-Macon, Emanuele Marchi, Nicholas P.J. Day, Ali Shojaie, Direk Limmathurotsakul, Narisara Chantratita, Paul Klenerman, Susanna J. Dunachie, T. Eoin West, Sina A. Gharib

**Affiliations:** 1 NDM Centre for Global Health Research, Nuffield Department of Clinical Medicine, University of Oxford, Oxford, United Kingdom.; 2 Mahidol-Oxford Tropical Medicine Research Unit, Salaya, Thailand.; 3 Department of Statistics and Probability, Michigan State University, East Lansing, USA.; 4 Department of Microbiology and Immunology, Mahidol University, Bangkok, Thailand.; 5 Sunpasitthiprasong Hospital, Ubon Ratchathani, Thailand.; 6 Division of Pulmonary, Critical Care and Sleep Medicine, University of Washington, Seattle, USA.; 7 Peter Medawar Building for Pathogen Research, University of Oxford, Oxford, United Kingdom.; 8 Department of Biostatistics, University of Washington, Seattle, USA.; 9 Translational Gastroenterology Unit, Nuffield Department of Clinical Medicine, University of Oxford, Oxford, United Kingdom.; 10 NIHR Oxford Biomedical Research Centre, Oxford University Hospitals NHS Foundation Trust, Oxford, Oxford, United Kingdom.; 11 Department of Global Health, University of Washington, Seattle, USA.

**Keywords:** Immunology, Infectious disease, Bacterial infections, Expression profiling

## Abstract

Melioidosis, a neglected tropical infection caused by *Burkholderia pseudomallei*, commonly presents as pneumonia or sepsis with mortality rates up to 50% despite appropriate treatment. A better understanding of the early host immune response to melioidosis may lead to new therapeutic interventions and prognostication strategies to reduce disease burden. Whole blood transcriptomic signatures in 164 patients with melioidosis and in 70 patients with other infections hospitalized in northeastern Thailand enrolled within 24 hours following hospital admission were studied. Key findings were validated in an independent melioidosis cohort. Melioidosis was characterized by upregulation of interferon (IFN) signaling responses compared with other infections. Mortality in melioidosis was associated with excessive inflammation, enrichment of type 2 immune responses, and a dramatic decrease in T cell–mediated immunity compared with survivors. We identified and independently confirmed a 5-gene predictive set classifying fatal melioidosis (validation cohort area under the receiver operating characteristic curve 0.83; 95% CI, 0.67–0.99). This study highlights the intricate balance between innate and adaptive immunity during fatal melioidosis and can inform future precision medicine strategies for targeted therapies and prognostication in this severe infection.

## Abstract 2

Despite appropriate treatment, melioidosis is associated with poor prognosis and high mortality. We reveal the complex host immune responses elicited by melioidosis and identify a 5-gene signature that can predict fatal cases.

## Graphical abstract

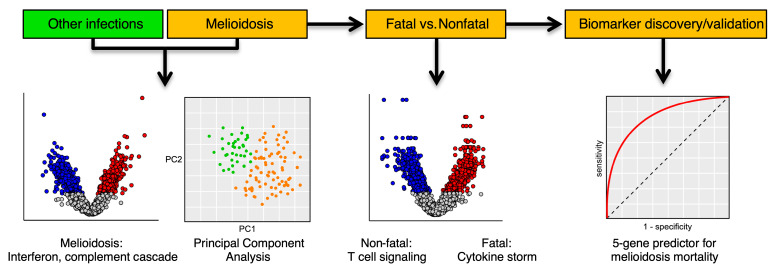

## Introduction

Melioidosis is a neglected tropical disease caused by the facultative intracellular Gram-negative bacterium and environmental saprophyte *Burkholderia pseudomallei*. The infection is highly endemic in Southeast Asia and in the Northern Territory of Australia, with hospitalized case fatality rates as high as 40% in Thailand but now less than 10% in Australia (1, 2). Recent mathematical modeling predicts that the global burden of melioidosis is underreported but is estimated to be 165,000 cases annually, of whom about 50% die (3). *B*. *pseudomallei* is increasingly detected around the world. For example, the pathogen was recently found in the environment and associated with locally acquired infection in the southern United States (4). Melioidosis is a common cause of community-acquired infection in northeastern Thailand and is usually associated with the presence of at least 1 risk factor such as diabetes mellitus or chronic kidney disease (5–7). The majority of melioidosis cases present to hospital with acute infection and the clinical presentations of disease are broad. However, pneumonia and bacteremia are the most common manifestations, often complicated by sepsis — i.e., life-threatening organ dysfunction caused by dysregulated host immune responses to infection (7–9).

The profound morbidity and mortality associated with melioidosis underscores the importance of acquiring a deeper understanding of the underlying pathophysiology and of developing new treatment strategies for the infection. We recently conducted a plasma metabolomic study of patients with melioidosis (10); however, profiling circulating leukocytes is an essential step in understanding the host immune response to this infection. Only a few studies have evaluated transcriptional perturbations in human melioidosis (11–14). In this study, we undertook the largest investigation to date of the whole blood transcriptome in melioidosis by studying patients with melioidosis and other infections who were prospectively enrolled within 24 hours after hospital admission. We tested the hypotheses that transcriptional signals in melioidosis are distinct from other infections, distinguish fatal from nonfatal cases, reveal mechanistic underpinnings of this infection, and can be leveraged to accurately predict death.

## Results

### Characteristics of the Ubon-sepsis discovery cohort.

The Ubon-sepsis discovery cohort comprised 234 patients with community-acquired infection including 164 individuals with culture-proven melioidosis, 35 with Gram-negative bacteremia other than *B*. *pseudomallei* (22 *E. coli* and 13 *Klebsiella pneumoniae*), 16 with Gram-positive bacteremia due to *Staphylococcus aureus*, and 19 who were blood culture negative. In addition, blood samples were obtained from 50 uninfected control individuals in the community (25 healthy blood donors and 25 patients with diabetes). The demographics and clinical characteristics of the participants are shown in Table 1 and Supplemental Table 1; supplemental material available online with this article; https://doi.org/10.1172/jci.insight.179106DS1 Compared with patients with other infections, those with melioidosis were younger, more likely to have diabetes, and experienced higher 28-day mortality. The modified Sequential Organ Failure Assessment (SOFA) score, a clinical assessment of organ failure, was higher in patients who died but was not different in patients with melioidosis versus other infections.

### IFN signaling and complement cascade activation distinguish melioidosis from other infections.

**Figure 1:**
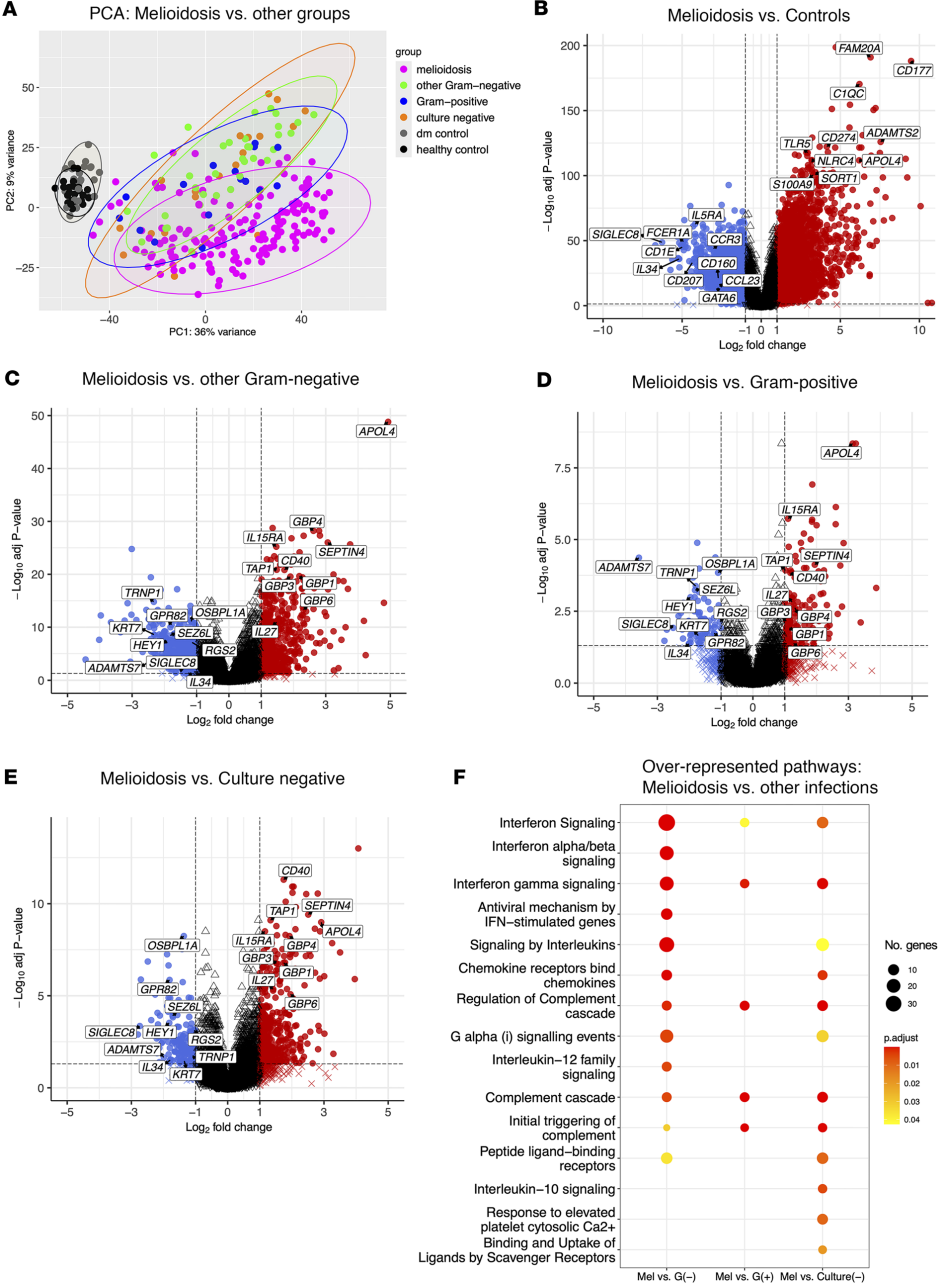
Differential gene expression and pathway analysis in patients with melioidosis and other infections in the Ubon-sepsis discovery cohort. (**A**) Principal component analysis (PCA) of the top 1,000 most variable genes in patients hospitalized with community-acquired infections and community control cohorts. Patients with infections are divided into melioidosis (melioidosis, magenta dots, *n* = 164), other Gram-negative infections (navy dots, *n* = 35), Gram-positive infection (cyan dots, *n* = 16), and negative bacterial culture (culture negative, coral dots, *n* = 19) groups. Uninfected nonhospitalized controls are divided into healthy controls (black dots, *n* = 25), and diabetic controls (dm control, gray dots, *n* = 25). Each ellipse represents 95% CI of bivariate t distribution. (**B**) Volcano plot of differentially expressed genes (DEGs) between patients with melioidosis and uninfected community controls. (**C**–**E**) Volcano plots of DEGs among melioidosis versus other Gram-negative infection groups (**C**), melioidosis versus Gram-positive infection groups (**D**), and melioidosis versus culture-negative patients (**E**). Red indicates upregulation and blue indicates downregulation of DEGs. Dotted lines define a cut-off of differentially expressed genes based on absolute log_2_ fold change ≥ 1 (*x* axis) and adjusted *P* < 0.05 (*y* axis). Selected DEGs are labeled. (**F**) Corresponding functional pathway analysis was performed among patients with melioidosis compared with patients hospitalized with other infections (other Gram negative: G[–]; Gram-positive: G[+]; culture negative: Culture[–]) based on Reactome gene sets. The top 10 overrepresented pathways are displayed. Overrepresentation analysis was performed on prefiltered upregulated DEGs based on a cut-off of absolute log_2_ fold change ≥ 1 and adjusted *P* < 0.05 derived from **C**–**E**. The gradient color bar corresponds to the adjusted *P* value. The size of each term is indicated by gene counts.

RNA-Seq was performed on whole blood obtained at the time of enrollment from the 284 participants. A total of 20,697 transcripts passed the filtering threshold. Principal component analysis (PCA) using the top 1,000 most variable genes showed clear segregation between the hospitalized infected patients and community control individuals as well as separation between patients with melioidosis and individuals hospitalized with other infections (Figure 1A). We performed step-wise differential gene expression analysis comparing the Ubon-sepsis cohort patients with melioidosis and uninfected controls. Since we found no differentially expressed genes (DEGs) between healthy and diabetic community controls (Supplemental Figure 2), we combined the 2 control subgroups into a single control cohort. Differential gene expression analysis identified 3,104 up- and 3,982 downregulated DEGs in patients with melioidosis compared with uninfected controls (adjusted *P* < 0.05, absolute log_2_ fold change ≥ 1) (Figure 1B), indicating widespread transcriptional changes. We then compared patients with melioidosis to patients with other Gram-negative infections (*E*. *coli* and *K*. *pneumoniae*), to patients with Gram-positive infection (*S*. *aureus*), or to patients with negative blood cultures. We identified substantial numbers of DEGs in patients with melioidosis compared with patients with other Gram-negative infections (486 up- and 510 downregulated DEGs), Gram-positive infection (139 up- and 86 downregulated DEGs), or the culture negative group (364 up- and 156 downregulated DEGs) (Figure 1, C–E). Functional pathway analysis of differentially upregulated genes in melioidosis versus all other infection groups, using Reactome gene sets, consistently identified overrepresentation of interferon (IFN) signaling and the complement cascade pathways in melioidosis (Figure 1F). These data establish that melioidosis induces dramatic transcriptomic perturbations in the human host and a gene expression profile that is distinct from other infections.

### Fatal melioidosis is associated with heightened innate immune activation but impaired T cell responses.

**Figure 2:**
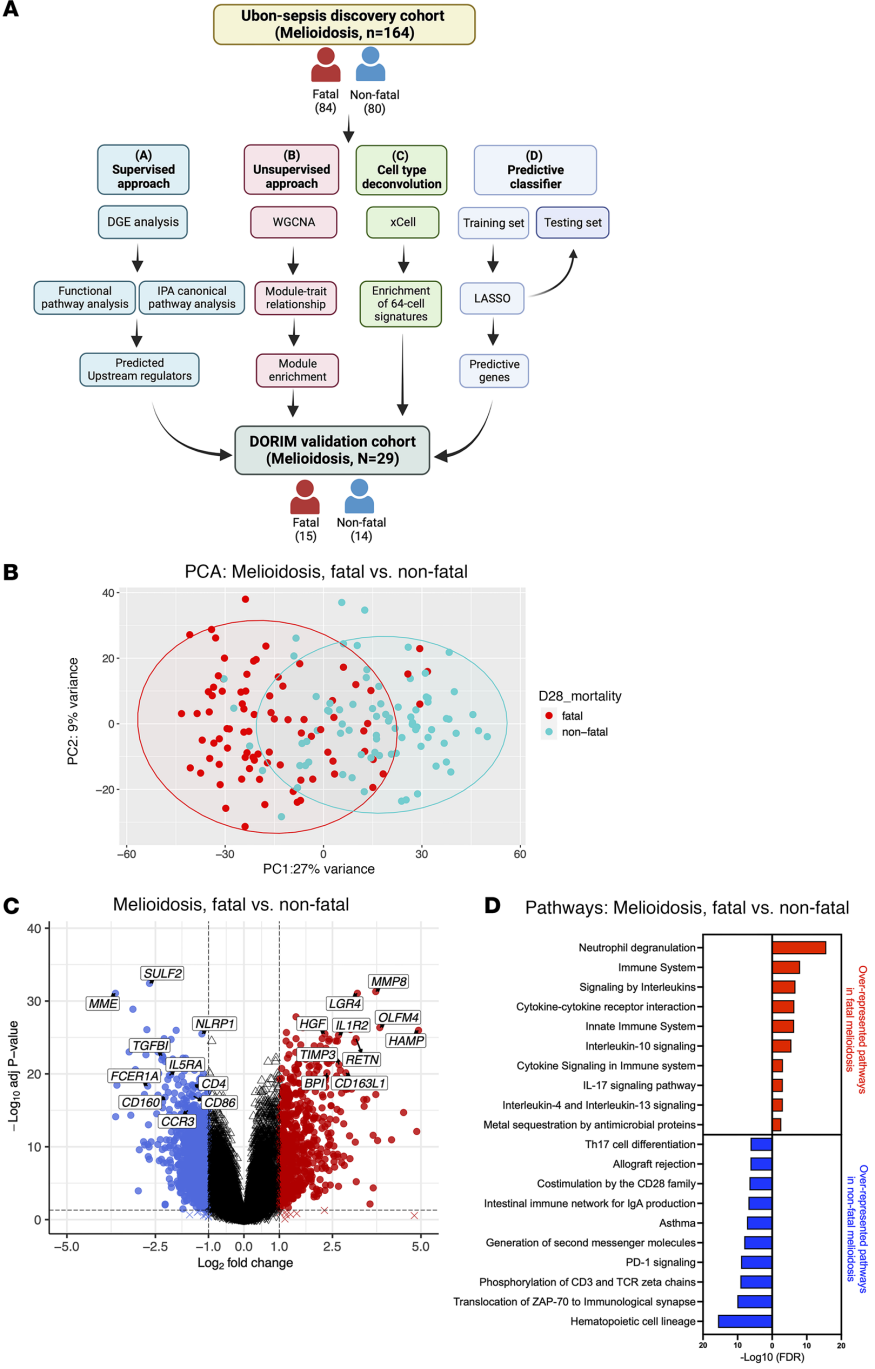
Transcriptomic profiling of fatal melioidosis in the Ubon-sepsis discovery cohort. (**A**) The core data analysis pipeline consists of 4 independent approaches: supervised data analysis, unsupervised data analysis, cell type deconvolution, and predictive classification. (**B**) Principal component analysis (PCA) of the top 1,000 most variable genes among patients with melioidosis in the Ubon-sepsis discovery cohort. Patients with melioidosis are divided into fatal (red dots, *n* = 84) and nonfatal (turquoise dots, *n* = 80) cases. Each ellipse represents 95% CI of bivariate t distribution. (**C**) Volcano plot of differentially expressed genes between fatal and nonfatal patients with melioidosis in the Ubon-sepsis discovery cohort. Dotted lines define a cut-off of differentially expressed genes based on absolute log_2_ fold change ≥ 1 (*x* axis) and adjusted *P* < 0.05 (*y* axis). Selected DEGs are labeled. (**D**) Corresponding overrepresentation analysis (ORA) between fatal and nonfatal patients with melioidosis in the Ubon-sepsis discovery cohort based on Reactome and KEGG gene sets. The top 10 overrepresented pathways are displayed by –log_10_ (FDR) values. ORA was performed on prefiltered upregulated DEGs based on a cut-off of absolute (log_2_ fold change ≥ 1) and adjusted *P* < 0.05. Created with BioRender.com.

Despite appropriate antimicrobial treatment, up to half of patients with melioidosis do not survive acute illness, underscoring the need to develop a better understanding of the adverse biological changes occurring during infection (3, 7, 15). We therefore adopted a multipronged analytic approach to identifying transcriptomic profiles associated with and predictive of 28-day mortality in melioidosis (Figure 2A). PCA on the patients with melioidosis within the Ubon-sepsis discovery cohort demonstrated separation of fatal and nonfatal melioidosis cases (Figure 2B). Differential gene expression analysis identified 633 up- and 1,109 downregulated genes in the fatal group compared with the nonfatal group (adjusted *P* < 0.05, absolute log_2_ fold change ≥ 1) (Figure 2C). People with diabetes are at a 12-fold increased risk of acquiring melioidosis (2); however, when we compared patients with melioidosis with and without diabetes, we did not find any DEGs, suggesting similar transcriptional responses once infection is established regardless of diabetes status (Supplemental Figure 2). Functional pathway analysis of DEGs in patients with melioidosis who died identified overrepresentation of pathways involved in inflammation such as neutrophil degranulation, innate immunity and cytokine interactions, IL-17 signaling, and type 2 immune responses — e.g., IL-10, IL-4, and IL-13 signaling (Figure 2D). Nonfatal melioidosis was strongly associated with overrepresentation of pathways involved in cellular immune responses by T cells (Figure 2D). Likewise, canonical pathway analysis by ingenuity pathway analysis (IPA) identified activation of IL-6 and cytokine/chemokine signaling, macrophage regulation, and T cell suppressive PD-1/PD-L1 pathways in fatal cases. T cell receptor signaling and IL-2 expression in T cells were very strongly suppressed (Table 2). To identify potential drivers of the observed transcriptional signals in melioidosis, we performed upstream regulator analysis using IPA and found that many of the most significant regulators were proinflammatory innate immune genes (Supplemental Table 2).

### Fatal melioidosis shares coexpressed gene modules with other markers of disease severity and key transcriptional regulators.

**Figure 3:**
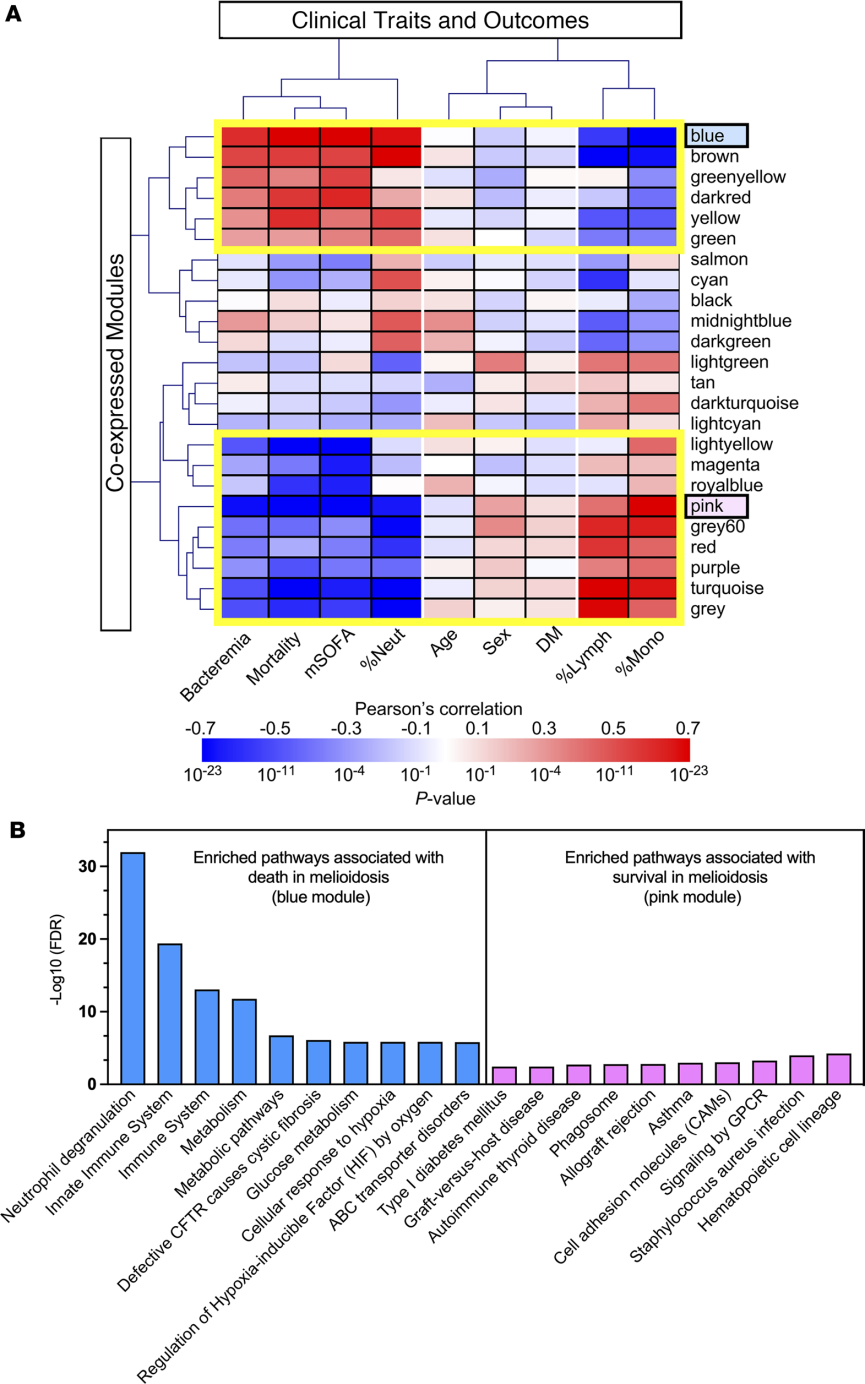
Module-trait relationship and module enrichment analysis by weighted gene coexpression network analysis in the patients with melioidosis in the Ubon-sepsis discovery cohort. (**A**) Heatmap of module-trait relationship in which each column and row represents clinical traits and module eigengenes. The gradient color bar indicates corresponding Pearson’s correlation coefficient and *P* value. In total, 161 participants with melioidosis from the Ubon-sepsis discovery cohort are included in the analysis. Clinical traits include age, sex, diabetes status (DM), 28-day mortality (mortality), bacteremia, percent neutrophils (%Neut), percent lymphocytes (%Lymph), and percent monocytes (%Mono). (**B**) Module enrichment analysis in module eigengenes (MEs) associated with death in melioidosis based on Reactome and KEGG gene sets. Module enrichment analysis was performed on the blue and pink modules shown in **A** with the highest correlations (positive and negative, respectively) with 28-day mortality status. The top 10 enriched pathways are displayed by –log_10_ (FDR) values.

We complemented our canonical pathway analyses with de novo pathway analysis using weighted gene coexpression network analysis (WGCNA) to identify 23 coexpressed modules associated with clinical outcomes in patients with melioidosis in an unbiased manner (Supplemental Figure 3). For ease of interpretation, each of these modules was assigned a color. Module-trait relationship analysis shows distinct correlation patterns between subsets of modules and clinical traits reflecting more severe disease (e.g., mortality, higher modified SOFA score, bacteremia, and greater percentage of blood neutrophils) (Figure 3A). In particular, the blue module eigengene (ME) was highly significantly correlated with mortality (Pearson’s rho = 0.64, *P* = 4 × 10^–20^). In contrast, the pink ME was the most significantly negatively correlated with mortality (Pearson’s rho = –0.56, *P* = 8 × 10^–15^).

We performed module enrichment analysis for these 2 modules (blue and pink) as representatives of the overall observed module-trait correlation patterns (Figure 3B). This analysis identified enriched inflammatory immune responses such as neutrophil degranulation and innate immune system activation in the blue module that was positively associated with mortality. In contrast, pathways involved in cellular immune processes such as hematopoietic cell lineage, signaling by GPCR, and allograft rejection were enriched in the pink module that was negatively associated with mortality.

### In deconvolution analysis, fatal melioidosis is associated with enrichment of innate immune cell signatures and reduction in cellular-mediated immune cell signatures.

**Figure 4:**
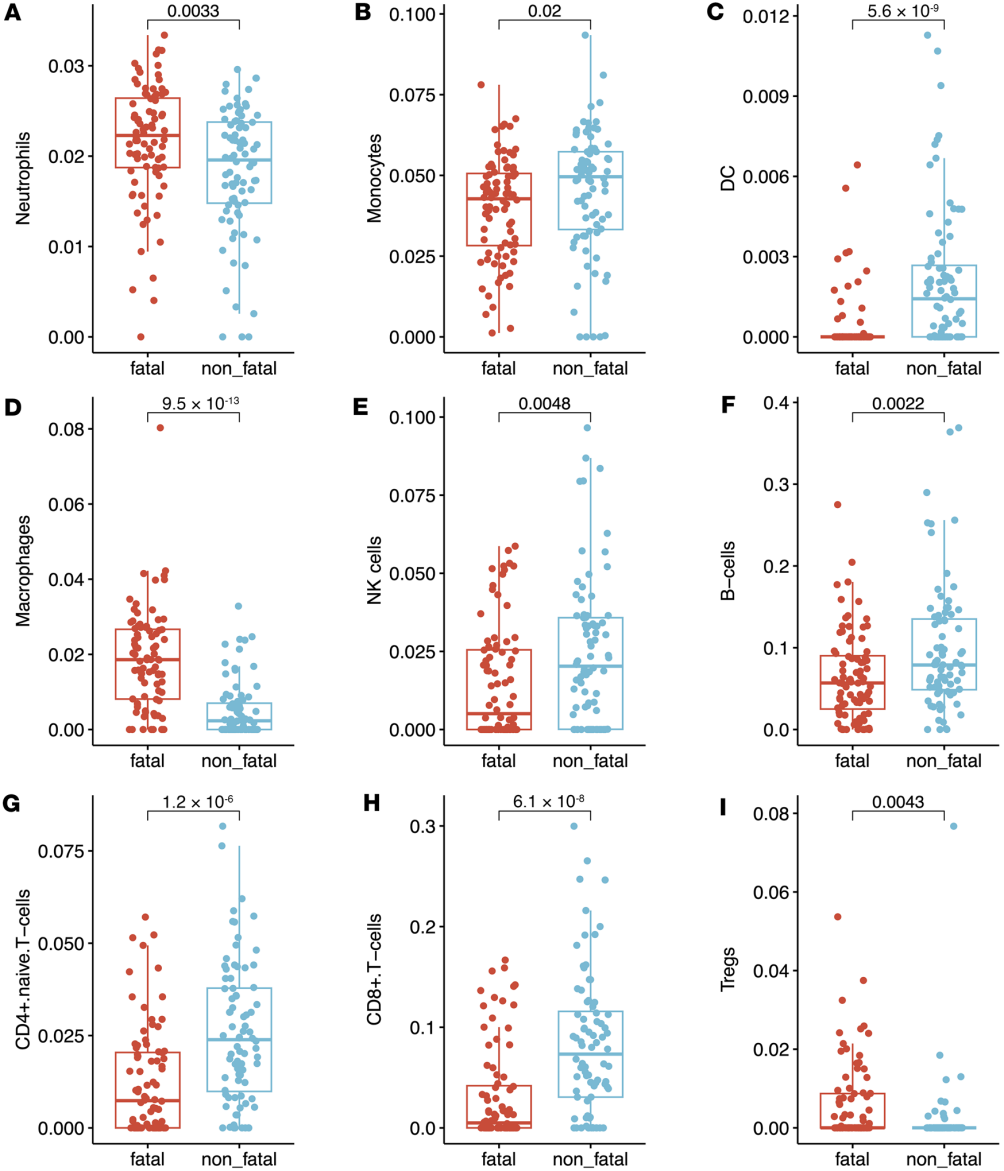
Imputed cell enrichment compared between nonsurvivors and survivors of melioidosis in the Ubon-sepsis discovery cohort. (**A**–**I**) The *y* axis displays enrichment score of each cell type. Nonparametric Mann-Whitney-Wilcoxon test was performed with its corresponding *P* value displayed on each plot alongside median and interquartile range boxes.

To estimate enrichment of different cell types in the whole blood transcriptomes, we applied xCell, a computational deconvolution tool, to our bulk RNA-Seq data (16). Of 64 cell signatures, 34 cell subsets were significantly different between fatal and nonfatal patients with melioidosis. Signatures characterizing neutrophils, Tregs, and macrophages were more enriched in the fatal cases (Figure 4). On the other hand, signatures for the majority of conventional T cells (both CD4 T and CD8 T cells), B cells, NK cells, monocytes, and DCs were more abundant in the nonfatal cases (Figure 4 and Supplemental Figure 4). These findings were concordant with the clinical full blood count differential measured upon patient enrollment that showed a greater percentage of neutrophils and lower percentages of lymphocytes and monocytes in fatal cases (Supplemental Figure 5).

### Validation of transcriptomic signatures, pathways, and modules in fatal melioidosis using an independent cohort.

To externally validate our findings, we applied the same differential gene expression, pathway, and WGCNA analytic approaches to whole blood transcriptomic data from an independent cohort of patients with melioidosis participating in a multicenter study (Determinants of Outcomes and Recurrent Infections in Melioidosis [DORIM]) performed at other sites in northeastern Thailand (*n* = 29; Supplemental Table 3) (12). Differential gene expression analysis identified 838 up- and 914 downregulated genes in the fatal (compared with the nonfatal) cases of the DORIM validation cohort, of which 188 up- and 261 downregulated genes were common to the Ubon-sepsis discovery cohort (Supplemental Figure 6). A comparative canonical pathway analysis revealed highly consistent enrichment patterns between the 2 cohorts, with suppression of several T cell–related pathways and activation of PD-1 and IL-6 signaling pathways in fatal melioidosis cases being particularly striking (Supplemental Table 4). Notably, 7 of the top 8 predicted upstream regulators (cytokines and transcription regulators) in fatal cases were shared between the 2 cohorts (Supplemental Table 5). The de novo pathway analysis using WGCNA similarly identified the ME blue module as having the highest correlation with 28-day mortality (Pearson’s correlation coefficient = 0.66, *P* = 1 × 10^–4^) (Supplemental Figure 7). The module enrichment analyses and deconvolution results of the validation study were largely comparable with those of the discovery cohort (Supplemental Figures 8 and 9). However, the deconvoluted signature of monocytes was lower in nonfatal cases from the DORIM validation cohort. Despite some differences, these validation analyses provided robust confirmation of our initial findings.

### Development and validation of a 5-gene predictive model for fatal melioidosis.

**Figure 5:**
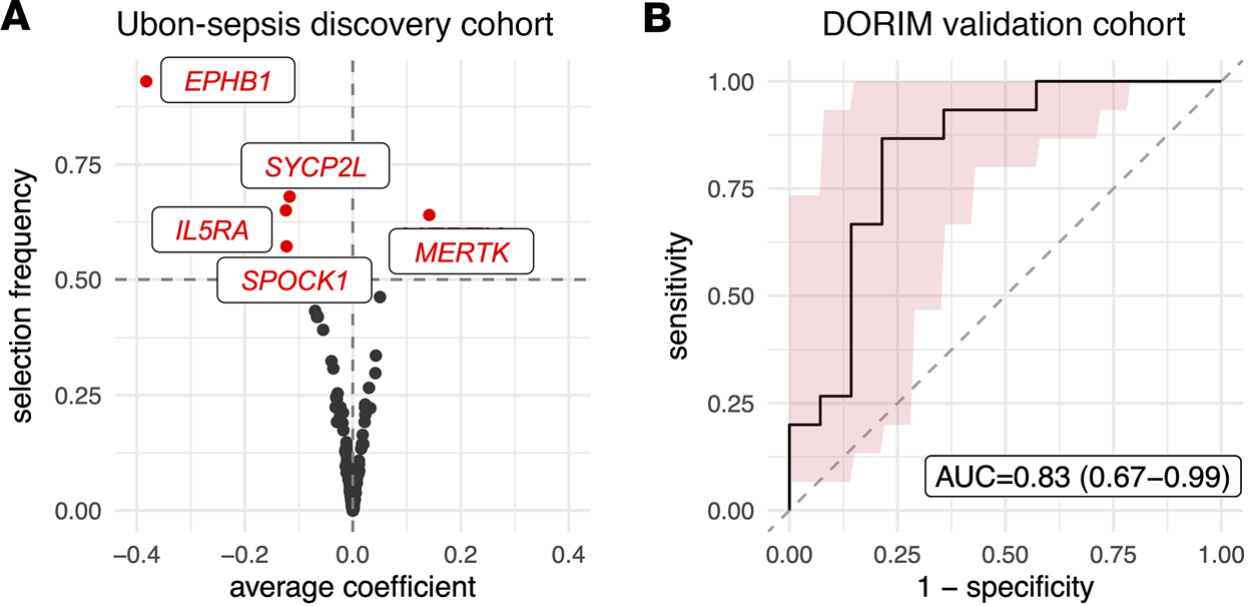
Top 5 predictive classifiers associated with fatal melioidosis. (**A**) Selection frequency and average coefficient for 2,000 transcripts using the repeated resampling strategy over 500 replications in the Ubon-sepsis discovery cohort. The 5 transcripts with > 50% selection frequency are labeled. (**B**) Receiver operating characteristic curve for predicting 28-day mortality on patients with melioidosis in the DORIM validation cohort based on the 5-transcript panel (shaded area indicates 90% confidence band).

We next selected 2,000 protein-coding transcripts with the largest variability from the Ubon-sepsis discovery cohort for predictive modeling of death in melioidosis. Using a Lasso-based repeated resampling strategy with 500 replications, we ranked these 2,000 transcripts based on their selection frequencies and average magnitudes of coefficients (Figure 5A). We created a model based on the 5 predictive genes (*EPHB1*, *SYCP2L*, *MERTK*, *IL5RA*, and *SPOCK1*) with selection frequency greater than 50%. We next validated the melioidosis mortality prognostic signature in the independent DORIM cohort, yielding an area under the receiver operating characteristic curve (AUC) of 0.83 (95% CI, 0.67–0.99) (Figure 5B). Sensitivity analyses comparing this 5-gene prognostic classifier with an organ failure score used for clinical prediction of critical illness mortality in DORIM demonstrated superior performance of the genetic predictor (Supplemental Table 6).

## Discussion

Melioidosis is an understudied tropical infection that causes a substantial global burden of disease, much of which is due to high mortality in endemic areas (17). In this comprehensive interrogation of the human blood leukocyte transcriptome, we show that melioidosis is associated with dramatic changes involving over a quarter of the transcriptome, reflecting the profound effect of the infection on the host. We also identified blood transcriptomic features that discriminate melioidosis from other community-acquired infections; most notably, blood transcriptomes of patients with melioidosis are dominated by IFN signaling and, to a lesser extent, complement cascade activation. Furthermore, we report distinctive transcriptomic profiles and gene modules associated with death in melioidosis. Patients who did not survive had marked activation of pathways involved in inflammatory innate immune responses concurrent with downregulation of genes related to T cell function compared with survivors. Lastly, we developed and validated a 5-gene predictive signature for death in melioidosis. Together, these observations expand our understanding of the pathophysiology in human melioidosis, identify potential immune cell–specific therapeutic targets, and demonstrate the promise of a predictive gene expression tool.

IFN responses, both type I and II, are a notable feature of the transcriptome in melioidosis. This observation is in agreement with a smaller gene expression study reporting IFN pathway activation in human melioidosis, our previous study showing higher plasma IFN-γ levels in melioidosis, and studies demonstrating the importance of IFN-γ in murine models of melioidosis (18–21). However, we also show that the IFN-dominated response in patients with melioidosis contrasts with patients with other infections who were recruited in an identical manner, underscoring the relative specificity of this profile in melioidosis among hospitalized patients with acute illnesses. IFN signaling is also reported in patients with tuberculosis, another intracellular infection, albeit one that tends to present as a chronic illness with much lower associated mortality (13, 22). The source of the IFN may be the adaptive T cell response or earlier cellular responses of cells such as NK and MAIT cells (23, 24).

Sepsis, the dysregulated host response to infection characterized by clinical evidence of organ failure, is a common clinical presentation of melioidosis. Despite the transcriptomic changes that discriminate melioidosis from other infections, sepsis is a syndrome that is diagnosed independently of type of infection. Even though there is substantial heterogeneity of sepsis (in host features, in infecting pathogens, and temporally), prior studies of transcriptional changes indicate that sepsis is often characterized by heightened neutrophil and pro-/antiinflammatory immune gene expression accompanied by decreased expression of adaptive immune responses (25, 26). Various transcriptomic endotypes reflecting activation of specific biological pathways have been derived that discern patients with sepsis at risk of poor outcomes and may help inform targeted clinical management (27–29). An attractive feature of our study is that, by focusing on infection from a single pathogen, we eliminate some of the heterogeneity within sepsis. Our complementary analyses of pathways associated with death in melioidosis, including overrepresentation analysis, WGCNA, and canonical pathway analysis, as well as our deconvolution studies were all concordant; patients with melioidosis who died (over 50% of patients in this study) had extreme upregulation of neutrophil degranulation and inflammatory cytokine pathways (including type 2 cytokines) concurrent with downregulation of T cell pathways. Interestingly, canonical pathway analysis (IPA) identified the PD-1/PD-L1 pathway, a well-known suppressor of T cell response, as being activated in fatal melioidosis despite downregulation of several gene members. This is consistent with a report showing suppression of T cell proliferation and signaling by polymorphonuclear cells infected with *Bps* through activation of PD-1/PD-L1 (30). We have also previously demonstrated that, in fatal cases of melioidosis, there is a failure of appropriate T cell activation in response to infection (31–33). The degree of immunosuppression characterizing fatal cases at the time of study enrollment is greater than that reported in a recent analysis of transcriptomic alterations associated with severity of illness or death in all-cause sepsis (29). This difference highlights the magnitude of a lethal dual hyperinflammatory/immunosuppressive pattern in melioidosis and suggests that these patients may benefit from targeted immunomodulatory therapies including the repurposing of PD-1/PD-L1 inhibitors (25, 34).

Prediction of patients at risk of poor outcomes is essential to inform clinical decisions about medical management, resource allocation, and referral, especially in locations with limited resources where many patients with melioidosis reside (35–37). There is a need for biological prediction tools to augment or replace clinical prediction methods. We therefore developed a 5-gene classifier to predict mortality in patients with melioidosis with good accuracy in an external validation cohort (AUC = 0.83), despite the smaller size, lower mortality, and later recruitment of patients in the validation set. To our knowledge, this is the first gene expression mortality prediction signature in melioidosis and, in the future, could be a useful method to help guide clinical decisions including immunomodulatory therapy administration.

Notably, we did not detect significant differences in the transcriptome of peripheral blood leukocytes between patients with diabets and those without diabetes in uninfected community controls or patients with melioidosis. This may be due to a true lack of difference, possibly inadequate sample size, or the cellular source of gene transcripts. We have previously shown differences in some circulating cytokines between patients with diabetes and those without diabetes in melioidosis as well as immune cell type–specific responses (32), but in this study, no significant signal was detectable at the gene expression level from pooled leukocyte populations (Supplemental Figure 2C). In addition, although diabetes is a key risk factor for melioidosis, consistent with previous studies, we did not find an association between diabetes and increased mortality in melioidosis (7, 38).

Our study has several strengths including a large discovery cohort of well-characterized patients with suspected infection who were enrolled systemically and prospectively over 4 years of study, the largest collection of melioidosis patient samples for RNA-Seq to date, and early enrollment of participants (with and without melioidosis) within 24 hours of admission to the study hospital. We also were able to validate our key findings in an independent cohort. However, there are several important limitations. We do not have protein level expression data to corroborate the gene expression results, although generating this is a goal. The validation cohort size was relatively small, and the enrollment strategy for this cohort required culture-confirmed melioidosis that may have delayed recruitment compared with the enrollment strategy for the discovery cohort. This approach may have missed some critically ill patients who died prior to enrollment and sampled patients later in illness. Lastly, the number of patients with infections other than melioidosis in the Ubon-sepsis discovery cohort was relatively small and underpowered for more in-depth analyses such as mortality.

In conclusion, the early transcriptomic signature in melioidosis is distinct from other infections, while fatal melioidosis is characterized by excessive host inflammatory immune responses coupled with impaired T lymphocyte responses. This highlights a likely pivotal role of the adaptive immune compartment in orchestrating successful immune resolution during melioidosis. These results can inform ongoing efforts to develop targeted precision medicine treatments for this severe infection.

## Methods

### Sex as a biological variable.

Our study examined both male and female participants.

### Study design and participants.

The discovery cohort is nested within the Ubon-sepsis cohort study which prospectively enrolled patients at least 18 years of age admitted to Sunpasittiprasong Hospital in Ubon Ratchathani, Thailand, with suspected community-acquired infection from 2013 to 2017 (5). Enrollment occurred within 24 hours of admission to the study hospital if patients demonstrated at least 3 documented systemic manifestations of infection as defined by the 2012 Surviving Sepsis Campaign (39). Blood was collected at the time of enrollment. Detailed clinical information was collected at serial time points during the hospitalization and at the time of discharge. Vital status was determined at 28 days. A modified SOFA score was calculated as described previously (5). For this study, a random sample of 164 patients with culture-confirmed melioidosis, defined by a positive *B*. *pseudomallei* culture of any clinical sample, was retrospectively identified. In addition, 22 patients with *E. coli* bacteremia, 13 with *K. pneumoniae* bacteremia, 16 with *S. aureus* bacteremia, and 19 who had negative blood cultures, all of whom were negative for *B*. *pseudomallei* infection, were randomly sampled from each infection group of the Ubon-sepsis cohort to serve as nonmelioidosis controls. These 234 individuals served as the discovery cohort. In addition, 25 nonhospitalized individuals with diabetes mellitus and 25 nonhospitalized individuals without any relevant medical conditions were recruited from the outpatient department and blood donation center at Udon Thani Hospital, Udon Thani province, Thailand, to serve as uninfected community controls. All participants were HIV^–^ at time of enrollment.

Validation of the findings from the Ubon-sepsis discovery cohort was performed by reanalyzing whole blood transcriptomic data generated in a cohort of patients with melioidosis that has been previously reported (12). Participants aged ≥ 15 years with culture-confirmed melioidosis were prospectively enrolled from a multicenter study (the DORIM study) between January 2015 and December 2019 at 7 provincial hospitals in northeastern Thailand (7, 21). Blood collected within 24 hours after culture of a clinical sample confirmed the diagnosis of melioidosis, a median of 3 days (range, 4–6 days) after hospital admission. RNA-Seq was performed on 29 individuals. These 29 individuals composed the DORIM validation cohort.

### Sample collection, isolation, and sequencing of total RNA.

Three milliliters of whole blood in Tempus Blood RNA tubes (Applied Biosystems) were obtained from participants in the discovery, control, and validation cohorts at the time of enrollment and frozen until further processing. For the discovery cohort and controls, total RNA from blood in Tempus Blood RNA tubes was extracted using the Tempus Spin RNA Isolation kit (Applied Biosystems). For the validation cohort, RNA isolation was performed using MagMAX for Stabilized Blood Tubes RNA Isolation Kit (Invitrogen). For the discovery cohort and controls, RNA-Seq was performed at the University of Washington Northwest Genomics Center. Poly-A libraries were sequenced on a NovaSeq6000 sequencer. For the validation cohort, libraries were sequenced using Ion PI Hi-Q Sequencing 200 Kit Chemistry (Invitrogen) on the Ion Proton System. Further details are provided in Supplemental Methods.

### Statistics.

Transcripts generated from sequencing were annotated to gene symbols. Genes with no counts or those expressed at low levels were removed. The overall data analysis pipeline plan is provided in Supplemental Figure 1. Initially, differential gene expression analysis (with age and sex as covariates) and functional pathway analysis were performed comparing patients with melioidosis in the discovery cohort to patients with other infections and community controls. Subsequently, a series of approaches was used to compare fatal versus nonfatal melioidosis cases (defined by vital status at 28 days) in the discovery cohort: differential gene expression analysis, pathway enrichment analysis methods with predefined pathways (overrepresentation analysis and canonical IPA), and de novo pathway analysis (weighted gene correlation network analysis) were used along with bulk RNA-Seq deconvolution (xCell) (16, 40–45). These approaches were also separately applied to the validation cohort of patients with melioidosis. Predictive modeling of 28-day mortality was performed on the patients with melioidosis within the discovery cohort. A repeated data resampling strategy, paired with lasso, was employed to select a robust panel of transcripts that best predicted mortality. The final panel of predictive transcripts from the patients with melioidosis in the discovery cohort was then tested on the validation cohort using leave-one-out prediction with penalized logistic regression, and a summary AUC was determined. Additional details about these methods are provided in the Supplemental Methods.

### Study approval.

Ethical approvals were obtained from the Ethics Committees/IRBs at Sunpasitthiprasong Hospital (039/2556); Udon Thani Hospital (6/2561); Mukdhahan Hospital (MEC 010/59); the Faculty of Tropical Medicine, Mahidol University (MUTM 2012-024-01, MUTM 2018-046-01, MUTM 2015-022-03); University of Washington (42988); and University of Oxford (OXTREC172-12). All participants gave written informed consent.

### Data availability.

Clinical and RNA-Seq data meeting Minimum Information About a Next-generation Sequencing Experiment (MINSEQE) guidelines have been deposited in dbGAP (accession no. phs003724.v1.p1). Values for all data points in graphs are reported in the Supporting Data Values file.

## Author contributions

Conceptualization was contributed by SJD, TEW, and SAG. Methodology was contributed by PR, LX, AS, and SAG. Formal analysis was contributed by PR and LX. Investigation was contributed by PR, LX; BK, TY, RP, AD, VH, GW, and LLM. Resources were contributed by DL, NC, and TEW. Writing of the original draft was contributed by PR. Review and editing of the manuscript were contributed by LX, BK, PC, EM, AS, DL, NC, PK, SJD, TEW, and SAG. Supervision was contributed by BK, PK, SJD, TEW, and SAG. Funding acquisition was contributed by NPJD, DL, NC, SJD, TEW, and SAG.

## Supplementary Material

Supplemental data

Supporting data values

## Figures and Tables

**Table 1:**
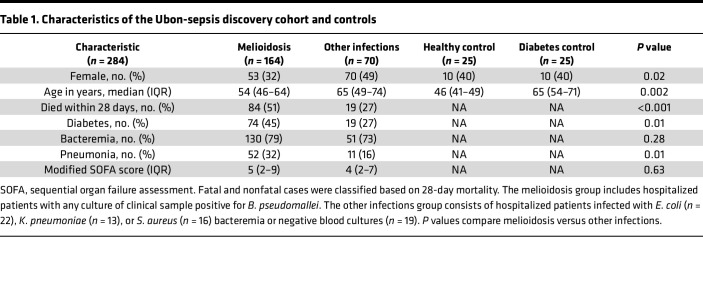
Characteristics of the Ubon-sepsis discovery cohort and controls

**Table 2:**
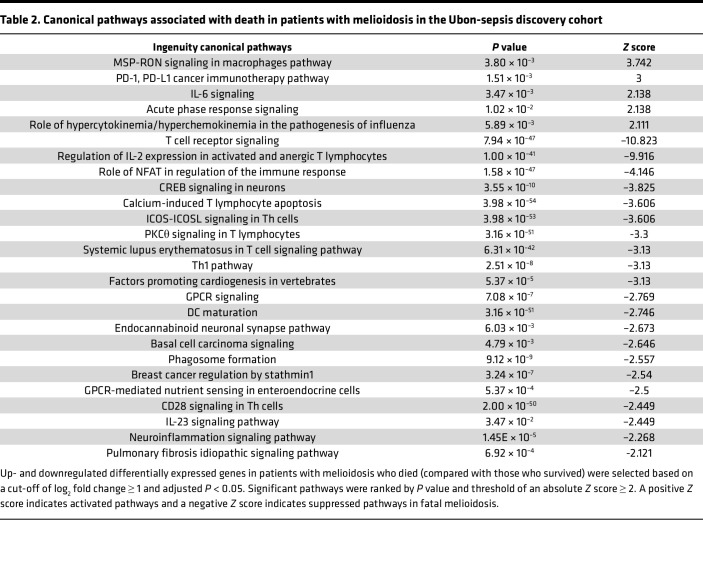
Canonical pathways associated with death in patients with melioidosis in the Ubon-sepsis discovery cohort

## Version 1 08/20/2024

In-Press Preview

## Version 2 09/24/2024

Electronic publication
