# Supplementary material for: Dysregulated immunologic landscape of the early host response in melioidosis
Source: JCI Insight. 2024 Aug 20;9(18):e179106. doi: 10.1172/jci.insight.179106 (PMC11457863; doi:10.1172/jci.insight.179106)
Supplement: Supplemental data [file jciinsight-9-179106-s248.pdf]

Supplemental Information

Dysregulated immunologic landscape of the early host response in melioidosis

Patpong Rongkard, Lu Xia, Barbara Kronsteiner, Thatcha Yimthin, Rungnapa Phunpang, Adul Dulsuk, Viriya Hantrakun, Gumphol Wongsuvan, Parinya Chamnan, Lara Lovelace-Macon, Emanuele Marchi, Nicholas P.J. Day, Paul Klenerman, Ali Shojaie, Direk Limmathurotsakul, Narisara Chantratita, Susanna J. Dunachie*, T. Eoin West*, Sina A. Gharib*

*Equal contribution

Table of Contents

Supplemental Materials and Methods, Page 3

Supplemental Figure 1, Page 8

Supplemental Figure 2, Page 10

Supplemental Figure 3, Page 11

Supplemental Figure 4, Page 12

Supplemental Figure 5, Page 14

Supplemental Figure 6, Page 16

Supplemental Figure 7, Page 17

Supplemental Figure 8, Page 18

Supplemental Figure 9, Page 19

Supplemental Table 1, Page 20

Supplemental Table 2, Page 21

Supplemental Table 3, Page 22

Supplemental Table 4, Page 23

Supplemental Table 5, Page 24

Supplemental Table 6, Page 25

Supplemental References, Page 26

Materials and Methods

Isolation of total RNA

For the discovery cohort and controls, total RNA from blood in Tempus Blood RNA tubes was extracted using the Tempus Spin RNA Isolation kit (Applied Biosystems). Frozen blood tubes were thawed prior to RNA extraction following the manufacturer's protocol. The isolated RNA was stored at -80◦C until analysis in elution buffers supplied within the RNA isolation kit. RNA yield and purity were determined with a Nanodrop ND-1000 spectrophotometer based on 260/280 and 260/230 ratios. For the validation cohort, RNA isolation was performed using MagMAX^{TM} for Stabilized Blood Tubes RNA Isolation Kit (Life technologies) and RNA concentration was measured using the NanoDrop Spectrophotometer (Thermo Fisher Scientific). The RNA quality was assessed using the Agilent RNA 6000 Pico kit on 2100 Bioanalyzer (Agilent Technologies) (*1*).

Whole blood RNA sequencing

For the discovery cohort and controls, RNA sequencing was performed at the University of Washington Northwest Genomics Center. Initial Quality Control (QC) entailed RNA quantification using the Quant-iT RNA assay (Invitrogen) and RNA integrity analysis using a fragment analyzer (Advanced Analytical). Poly-A library preparation from total RNA was performed using the TruSeq Stranded mRNA kit as outlined by the manufacturer (Illumina, cat#RS-122-2103). Libraries were sequenced on a NovaSeq6000 sequencer. For the validation cohort, RNA libraries were prepared using Ion AmpliSeq^{TM} Transcriptome Human Gene Expression Kit (Thermo Fisher Scientific). Libraries were sequenced using Ion PI Hi-Q Sequencing 200 Kit chemistry (Life Technologies) on the Ion Proton System (*1*).

Upstream RNA sequencing data analysis

For the discovery cohort and controls, raw RNA-sequencing data (FASTQ files) were aligned to GRCh38 with reference transcriptome GENCODE release 30 using STAR (v2.6.1d). Gene level expression quantification was generated with RNA-SeQC (v2.3.3) and RSEM (v1.3.1). For the validation cohort, the final libraries were loaded onto Ion PI v3 chips and sequenced using Ion PI Hi-Q Sequencing 200 Kit chemistry (Life Technologies) on the Ion Proton System for 200 bp read length (*1*).

Core bioinformatics approaches

Supervised and unsupervised learning approaches and bulk RNA-sequencing deconvolution were first applied to patients in the discovery (Ubon-sepsis) cohort (Supplemental Figure 1). These approaches were also separately applied to the validation (DORIM) cohort of melioidosis patients. In addition, a mortality prediction signature was developed to classify the melioidosis patients in the discovery cohort and this was subsequently tested in the validation cohort. (Supplemental Figure 1).

I. Differential gene expression and functional pathway analysis

For the discovery cohort and controls, a total of 58,673 transcripts were generated from the sequencing and annotated to gene symbol (HUGO Gene Nomenclature Committee). Genes with no counts or those expressed at low levels were removed. Remaining filtered genes were normalized and variance stabilizing transformed for down-stream data analysis and data visualization using DESeq2 R package (*2*). For the validation cohort, a total of 20,812 transcripts were generated from the sequencing and annotated to gene symbol; these were processed similarly. To assess whether melioidosis induces global transcriptional responses, we performed dimension reduction by principal component analysis using the top 1,000 most variable genes in the melioidosis and control subjects. Differential gene expression analysis was carried out using negative binomial generalized linear model implemented in DESeq2 R

package (version 1.34.0) controlling for age and sex as covariates. The false discovery rate (FDR) was used to correct for multiple comparisons, in which an FDR-adjusted P-value <0.05 was deemed significant unless stated otherwise (*3*). Functional pathway analysis was performed on differentially expressed genes (DEGs) with FDR <0.05 and absolute(Log$_2$ fold change)1. Functional pathway analysis based on Reactome and KEGG databases was performed using WebGestalt (http://www.webgestalt.org/) or clusterProfiler R package (version 4.0.5), in which overrepresented pathways with FDR <0.05 were deemed significant (*4, 5*). Additionally, Ingenuity Pathway Analysis (IPA) was used to identify significant canonical pathways and upstream regulators (P-value <0.05, absolute[z-score]2) on differentially up- and down- regulated genes. To examine the impact of case fatality based on 28-day mortality status on whole blood transcriptome, we applied identical data analysis approaches including differential gene expression analysis and pathway analyses in comparison between fatal and non-fatal melioidosis patients.

II. Weighted gene correlation network analysis (WGCNA)

In parallel with the above analyses, we performed data-driven, agnostic analysis of all RNA-seq data without inclusion of any *a priori* information using WGCNA. Co-expressed gene networks were used to identify significant gene module and clinical trait relationships and enriched pathways within the significant gene modules. The pre-filtered, normalized and variance stabilizing transformed expression data (19,919 features) of the melioidosis patients in the discovery cohort (n=162) were entered into WGCNA R package (version 1.70-3) (*6*). For the validation cohort, WGCNA was performed on pre-filtered 16,532 features, following normalized and variance stabilizing transformed expression data of 29 melioidosis patients in the validation cohort. First, a sample dendrogram was constructed to detect potential outliers using the average linkage method. A signed co-expression network analysis was performed with default settings, unless stated otherwise. Second, a soft power was chosen to achieve a

scale-free network. Third, a cluster dendrogram of co-expressed gene modules was constructed by hierarchical clustering of genes based on a dissimilarity measure (1-topological overlap matrix, 1-TOM), resulting in co-expression modules and module eigengenes (MEs) distinguished with different colors. Fourth, the co-expression modules were further studied for their relationships with clinical traits (module-trait relationship analysis) based on Pearson's correlation. The modules of interest with significant module-trait relationships underwent pathway enrichment analysis based on Reactome and KEGG databases using an extended Bioconductor package for WGCNA, anRichment (*6*).

III. Deconvolution of bulk RNA sequencing data using xCell

Cell type enrichment was estimated from the bulk RNA sequencing data using single sample gene-set enrichment analysis (ssGSEA) method by xCell (*7*). First, read counts were normalized to Reads Per Kilobase of transcript per Million reads mapped (RPKM) using edgeR R package (*8*). Here, a 64-cell signature such as lymphoid, myeloid, and stem cells was performed using xCell R package (version 1.1.0). Samples with P-value <0.05 were included for further analyses. The enriched cell types between fatal and non-fatal melioidosis patients were compared using Mann-Whitney-Wilcoxon test in R.

IV. Constructing and validating a predictive model for fatal melioidosis

To facilitate the prediction of 28-day mortality among patients with melioidosis, the Ubon-Sepsis discovery cohort of 164 melioidosis patients were used to generate a parsimonious model. Variance stabilizing transformation was applied to the expression data (R package "DESeq2"), followed by standardization to z-scores. Transcripts with a coefficient of variation greater than 0.25 were filtered. A repeated data resampling strategy, paired with LASSO, was employed to select a robust panel of transcripts that best predicted mortality. Each time, 70% of the training data were randomly selected without replacement, based on which penalized logistic regression was applied for variable selection (R package "glmnet").

The transcripts were ranked based on their average selection frequencies and magnitudes of coefficients over 500 times of repetitions and those with selection frequency above 50% were selected for independent validation (Figure 5A). This 5-gene panel of predictive transcripts from the Ubon-sepsis discovery cohort of melioidosis patients was then tested on the DORIM validation cohort of 29 melioidosis patients. The transcriptomic data from the validation cohort were normalized and transformed in the same manner as the Ubon-sepsis discovery cohort. Leave-one-out prediction with penalized logistic regression was performed on DORIM and the area under the receiver operating characteristic curve (AUC) was summarized (R package "pROC"). We performed additional sensitivity analyses to assess the performance of the 5-gene panel in DORIM compared to an organ failure score. This score was calculated using the respiratory, cardiovascular, liver, renal, and coagulation measurements from a SOFA score as we have previously described (*9*). In Supplemental Table 6, we applied several logistic regression models (Model 1: organ failure score only, Model 2: Model 1 + age, sex and diabetes) coupled with leave-one-out cross-validation to predict mortality in the validation cohort (DORIM), yielding areas under the receiver operating characteristic curve (AUC) of 0.695 and 0.652 for the two logistic regression models above, as compared to AUC = 0.833 for the 5 gene prognosticator (Model 3). Therefore, the gene classifier outperforms an organ failure clinical predictor in the DORIM validation cohort.

Supplemental Figure 1

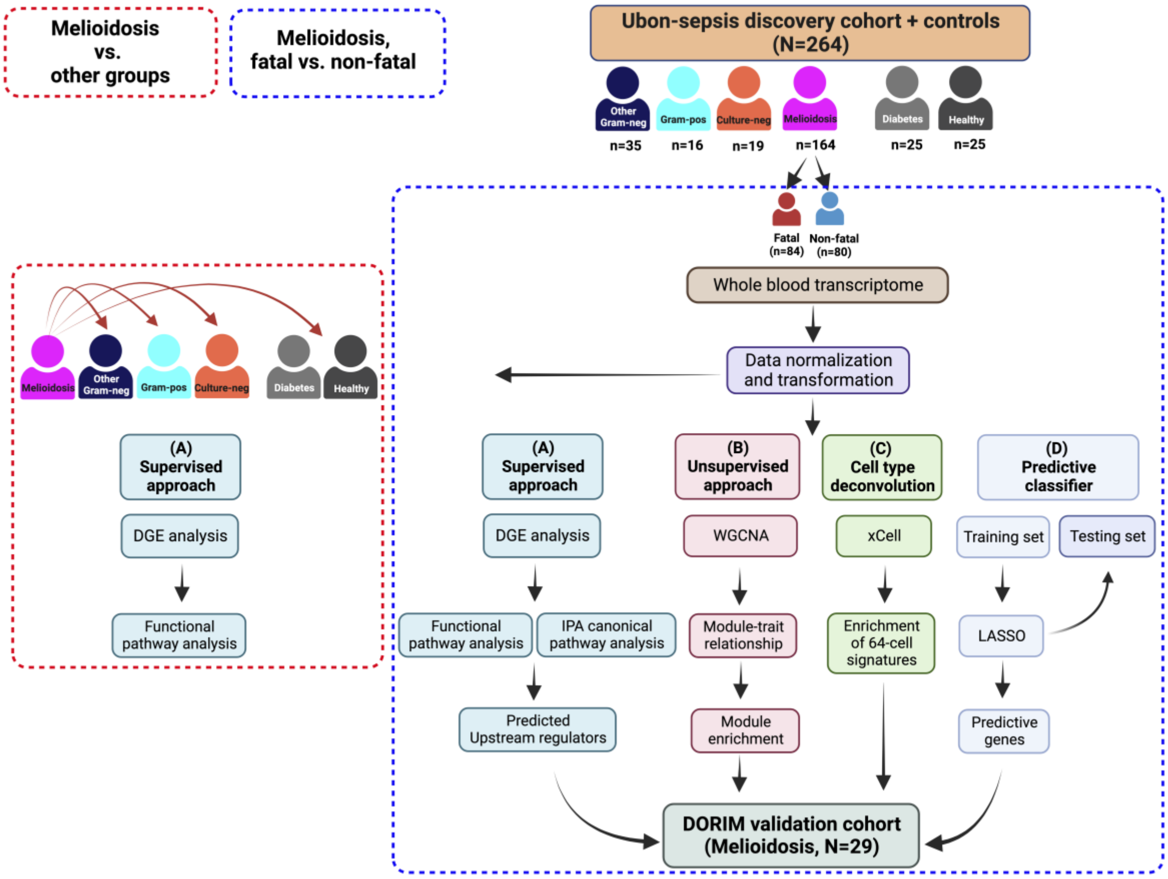

Supplemental Figure 1. Flowchart of data analysis pipelines for (i) characterization of blood transcriptomic profiles associated with melioidosis compared to other community-acquired infections (red dotted square) and (ii) identification of blood transcriptomic profiles associated with fatal melioidosis (blue dotted square). Core data analysis pipeline consists of four independent approaches; supervised, and unsupervised data analysis, cell type deconvolution, and predictive classification. A: the supervised approach employs a differential gene expression analysis using DESeq2 and followed by pathway analyses and prediction of upstream regulators by ingenuity pathway analysis (IPA). B: the unsupervised approach uses weighted gene co-expression network analysis (WGCNA) and followed by module-trait relationship and module enrichment analysis. C: the cell type deconvolution technique uses

xCell method for enrichment of 64-cell signatures. D: the predictive classifier employs the Least Absolute Shrinkage and Selection Operator (LASSO) regression model to identify predictor genes distinguishing fatal from non-fatal melioidosis patients. The Ubon-sepsis discovery cohort consists of 164 melioidosis patients and 70 patients with other community-acquired infections including other Gram-negative infections (“Other Gram-neg”, n=35), Gram-positive infection (“Gram-pos”, n=16), and negative bacterial culture (“Culture-neg”, n=19). The DORIM validation cohort consists of 15 fatal and 14 non-fatal melioidosis patients.

Supplemental Figure 2

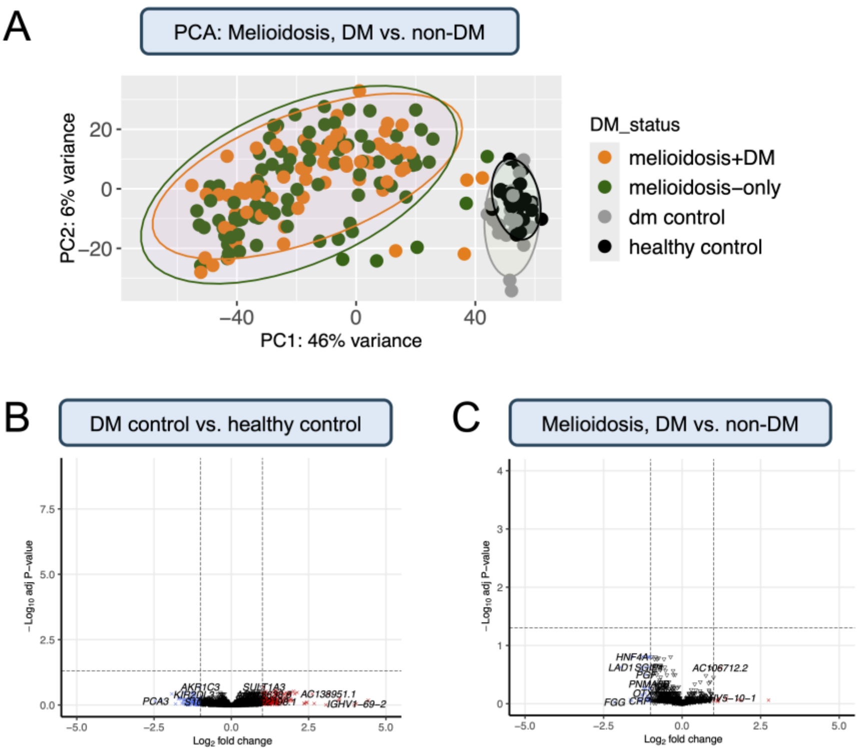

Supplemental Figure 2. Principal component analysis (PCA) and differential gene expression analysis between melioidosis with and without diabetes. A: PCA of the top 1,000 most variable genes in melioidosis and control cohorts colored by diabetes status (DM_status). Patients with melioidosis, divided into diabetes ("melioidosis+DM", orange dots, n=84), non-diabetes ("melioidosis-only", green dots, n=80), healthy controls ("healthy control", black dots, n=25), and diabetic controls ("dm control", grey dots, n=25). B: Volcano plot of differentially expressed genes (DEGs) between diabetic controls (n=25) and healthy controls (n=25). C: Volcano plot of DEGs between melioidosis patients with diabetes (n=84) and without diabetes (n=80). Dotted lines define a cut-off of DEGs based on absolute (Log_2 fold-change)≥ 1 (x-axis) and adjusted P-value < 0.05 (y-axis).

Supplemental Figure 3

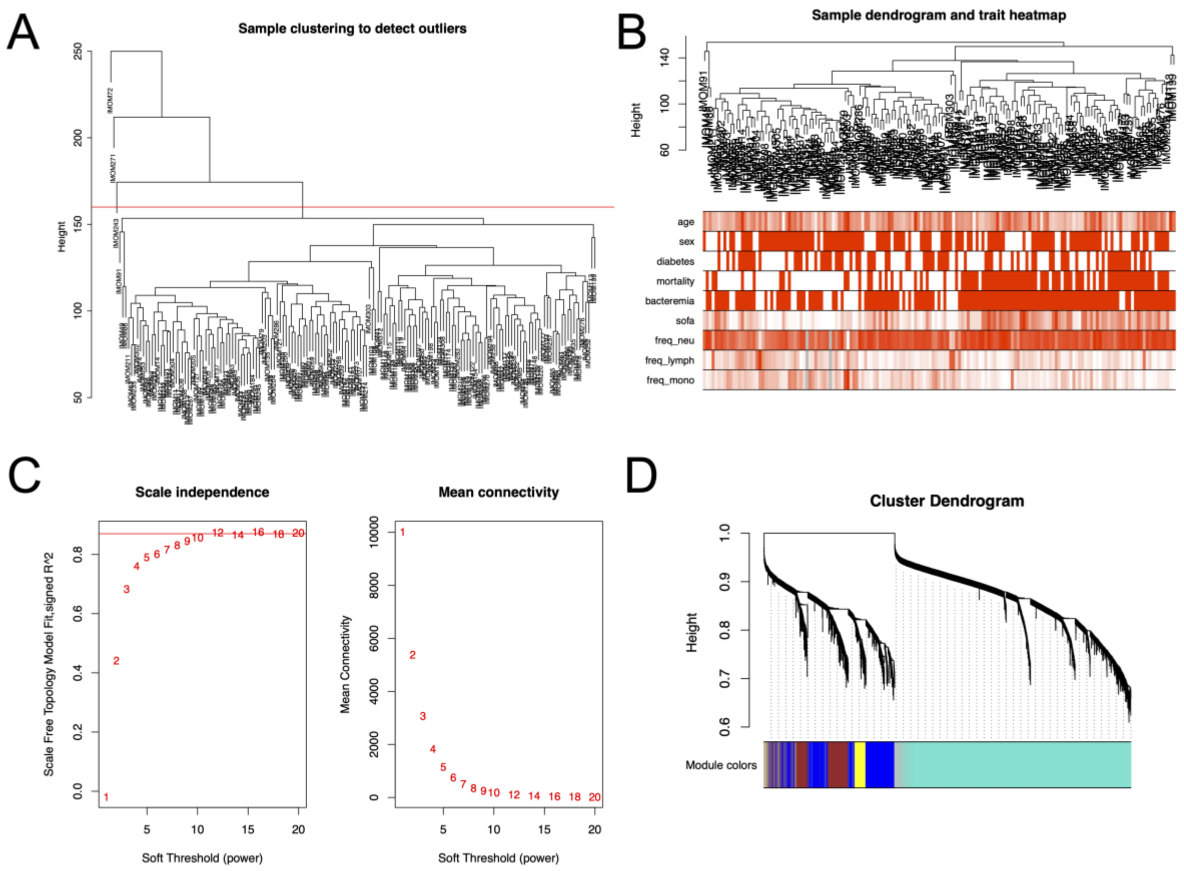

Supplemental Figure 3. Weighted gene co-expression network analysis workflow.

A: A sample dendrogram detected three outliers. B: A sample dendrogram with corresponding trait heatmap after removing the outliers. C: Scale-free network analysis with scale independence plot indicates scale free topology model fit (R^2) and mean connectivity plot with soft threshold on x-axis. A power of b = 12 was chosen, yielding a soft-threshold $R^2 = 0.87$ and mean connectivity = 110. D: Cluster dendrogram of co-expressed gene modules by hierarchical clustering of genes based on topological overlap with assigned module colors underneath.

Supplemental Figure 4

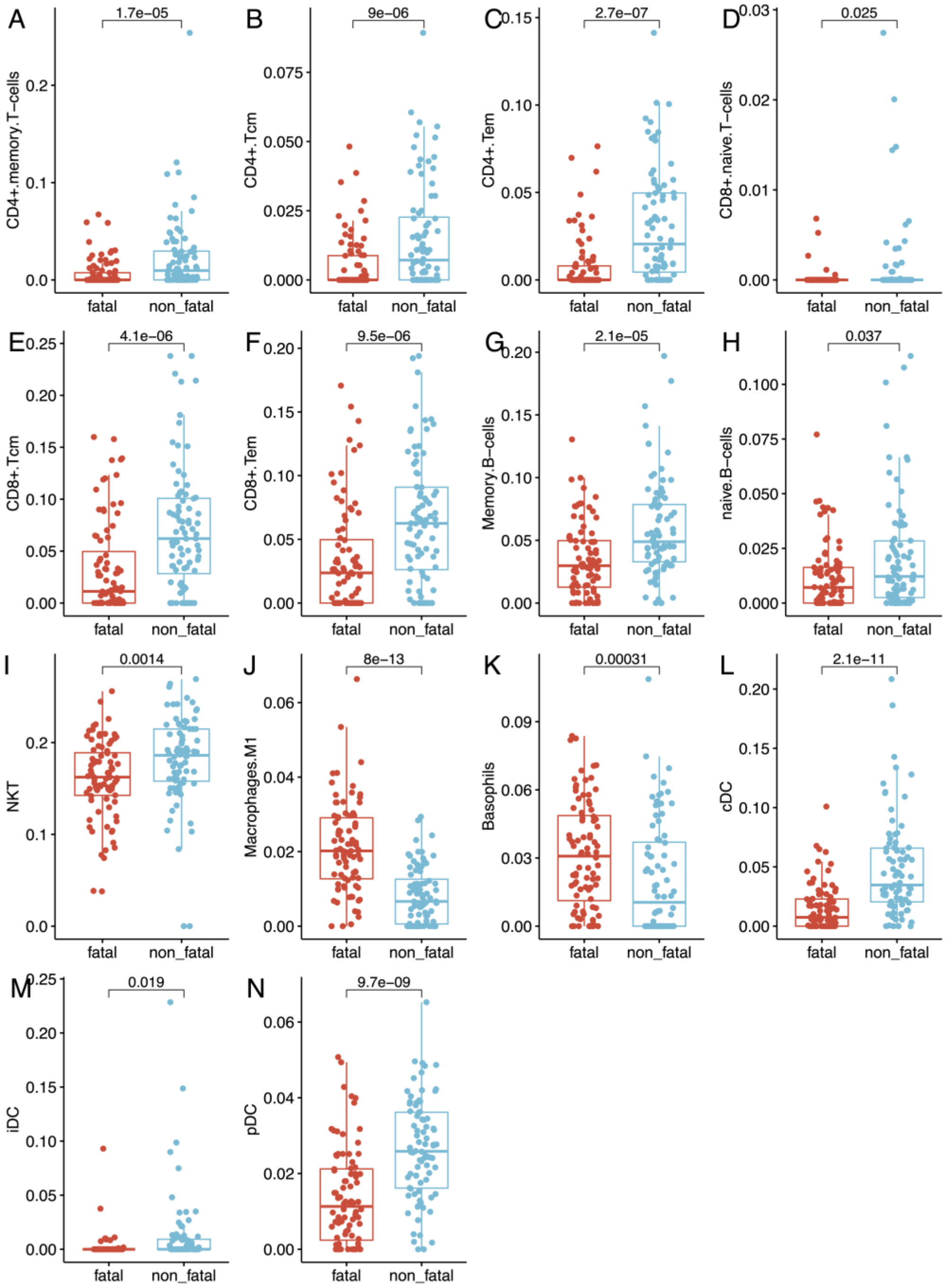

Supplemental Figure 4. Scattered boxplots show a subset of imputed cell enrichment compared between non-survivors and survivors of melioidosis in the Ubon-sepsis discovery cohort including all remaining differential myeloid and lymphoid populations not displayed in Figure 4. The Y-axis displays enrichment score of each cell type. Non-parametric Mann-

Whitney-Wilcoxon test was performed with its corresponding P-value displayed on each plot alongside median and inter-quartile range boxes.

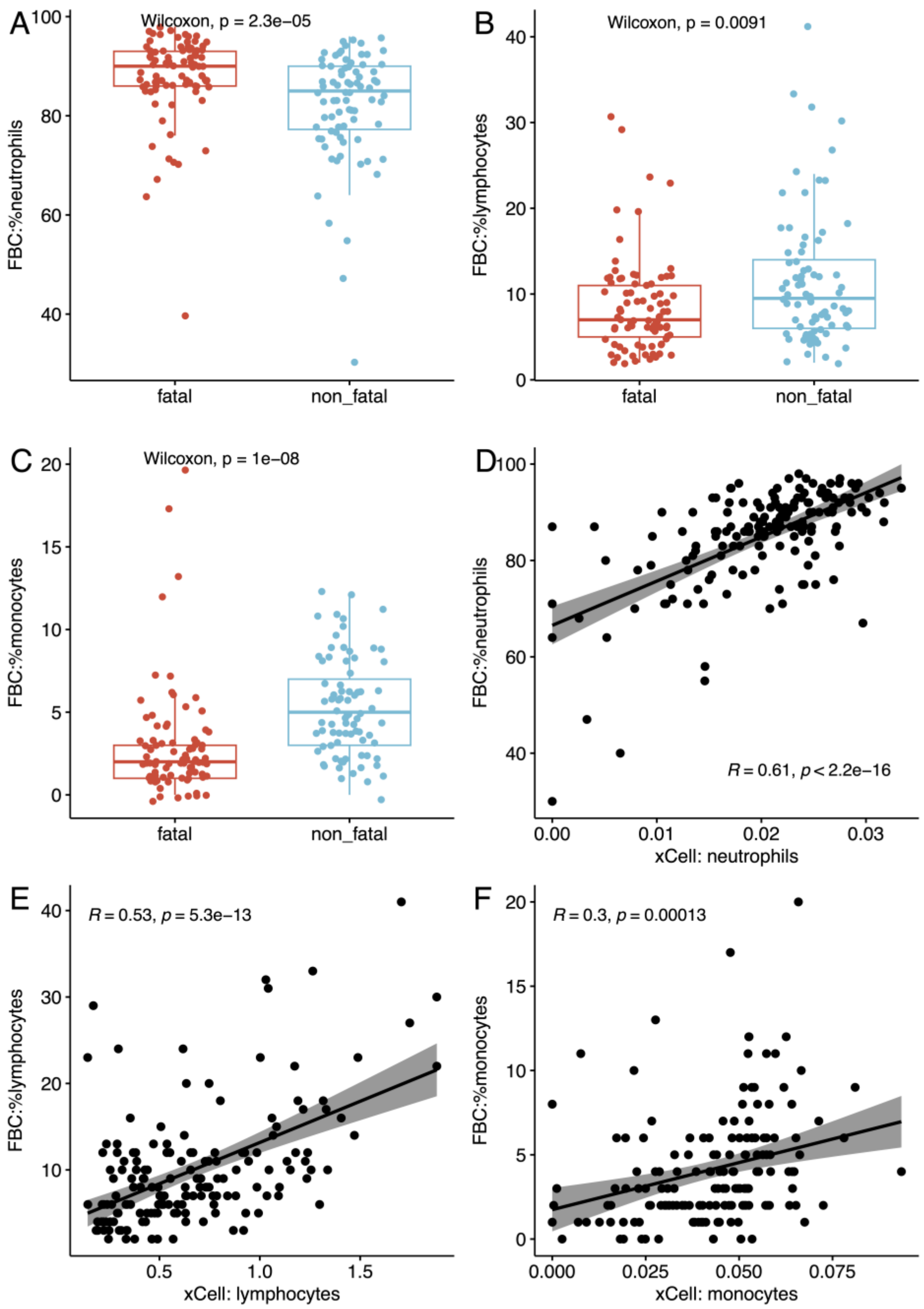

Supplemental Figure 5. Scatter plots show the frequencies of neutrophils, lymphocytes, and monocytes from the full blood count (FBC) upon enrolment of melioidosis patients in the Ubon-sepsis discovery cohort and correlations between deconvoluted cell enrichment scores by the xCell method (x-axis) and the results of FBC (y-axis). (A-C) The results by FBC were compared between fatal and non-fatal melioidosis patients using non-parametric Mann-Whitney test with its corresponding P-value displayed on each plot along with median and

inter-quartile range bars. (D-F) For the correlation analysis, frequencies of each white blood cell were correlated with representative profiles of deconvoluted immune cell type using Pearson's correlation test. Corresponding Pearson's correlation coefficient, P-value and linear regression line with 95% confidence interval (shaded area) are displayed within each plot.

Supplemental Figure 6

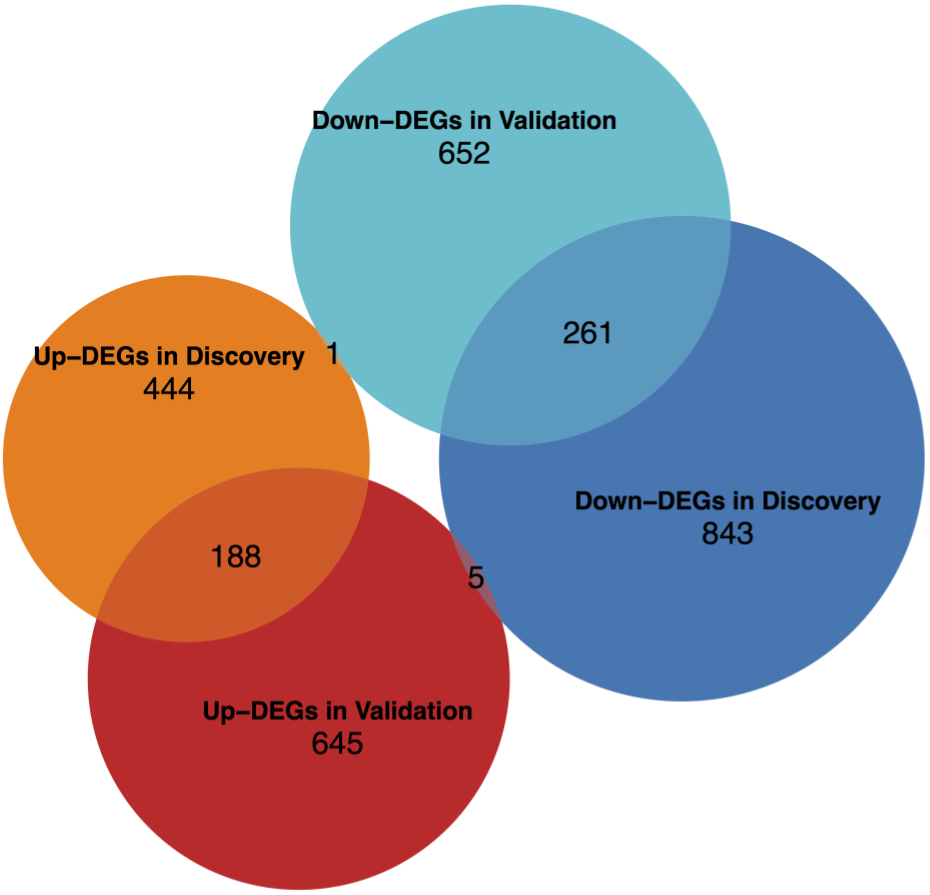

Supplemental Figure 6. Overlapping differentially expressed genes (DEGs) distinguishing fatal from non-fatal melioidosis cases in the Ubon-sepsis discovery cohort and in the DORIM validation cohort. Significant DEGs are based on absolute (log_2 fold-change)1 and adjusted P-value <0.05.

Supplemental Figure 7

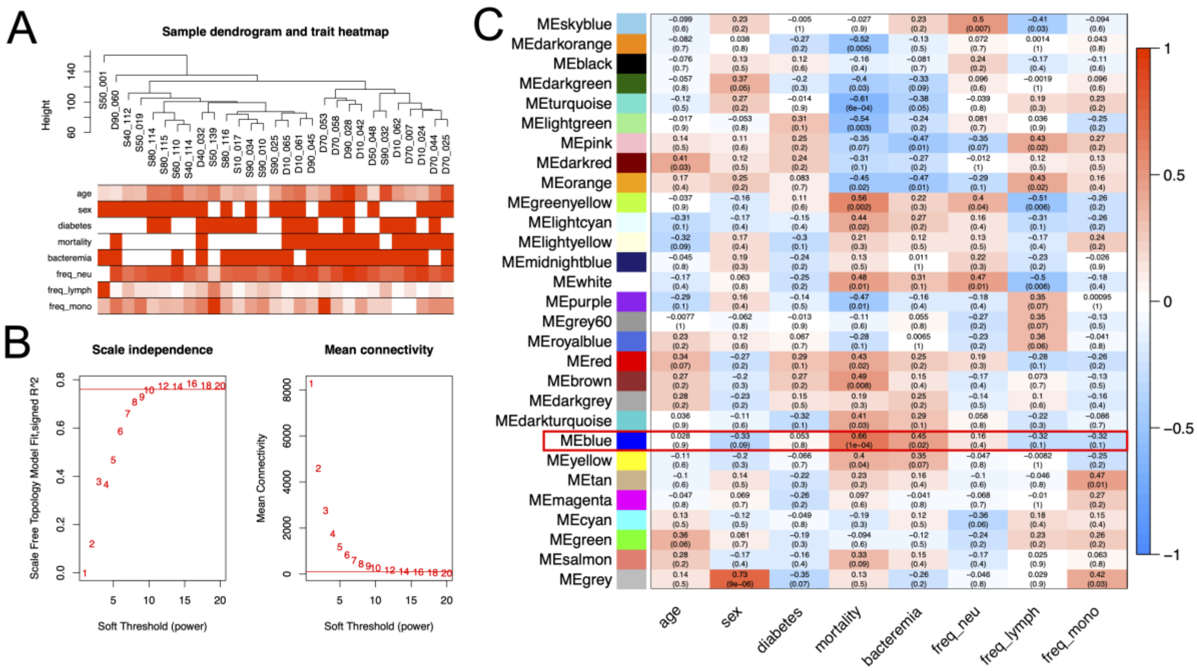

Supplemental Figure 7. Weighted gene co-expression network analysis workflow of 28 melioidosis patients in the DORIM validation cohort. A: Sample dendrogram with corresponding trait heatmap. B: Scale-free network analysis with scale independence plot indicates scale free topology model fit (R^2) and mean connectivity plot with soft threshold on x-axis. A power of b = 14 was chosen, yielding a soft-threshold R^2 = 0.77 and mean connectivity = 121. C: Module-trait relationship analysis in melioidosis by weight gene co-expression network analysis. Each column and row represents clinical traits and module eigengenes, respectively, with corresponding Pearson's correlation coefficient and P-value in parenthesis. A gradient color bar indicates positive and negative Pearson's correlation coefficient. Clinical traits include age, sex, 28-day mortality (mortality), diabetes status (diabetes), bacteremia, %neutrophils (freq_neu), and %lymphocytes (freq_lymph), and %monocytes (freq_mono).

Supplemental Figure 8

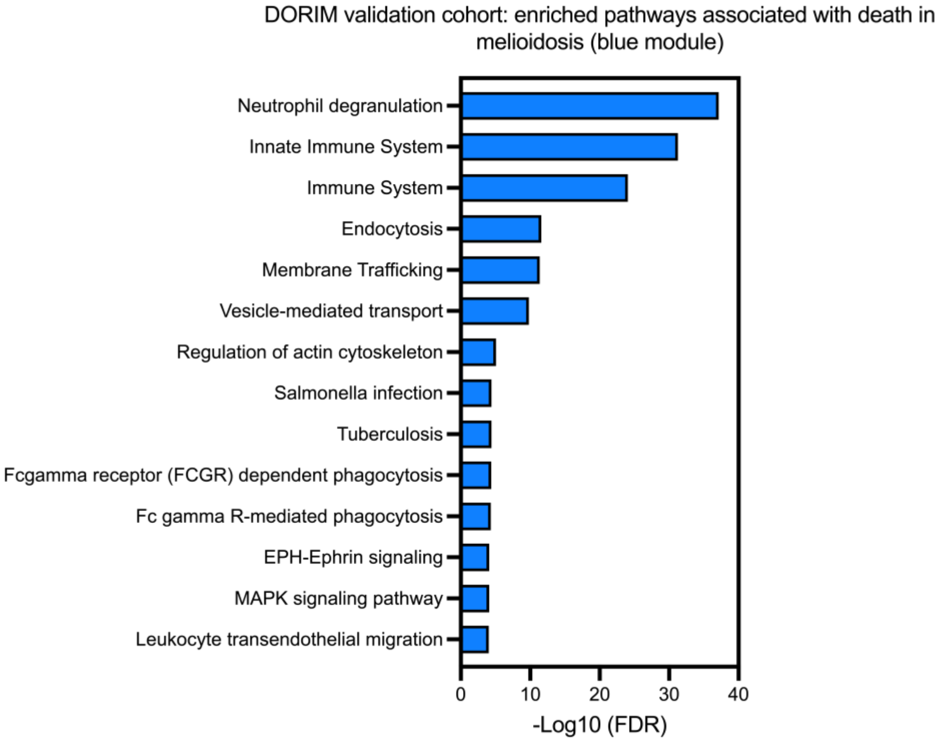

Supplemental Figure 8. Enrichment analysis based on Reactome and KEGG gene sets in ME Blue module which were highly associated with mortality identified by module-trait relationship analysis in 28 melioidosis patients in the DORIM validation cohort. Enriched pathways are displayed by $-\log_{10}$ (FDR).

Supplemental Figure 9

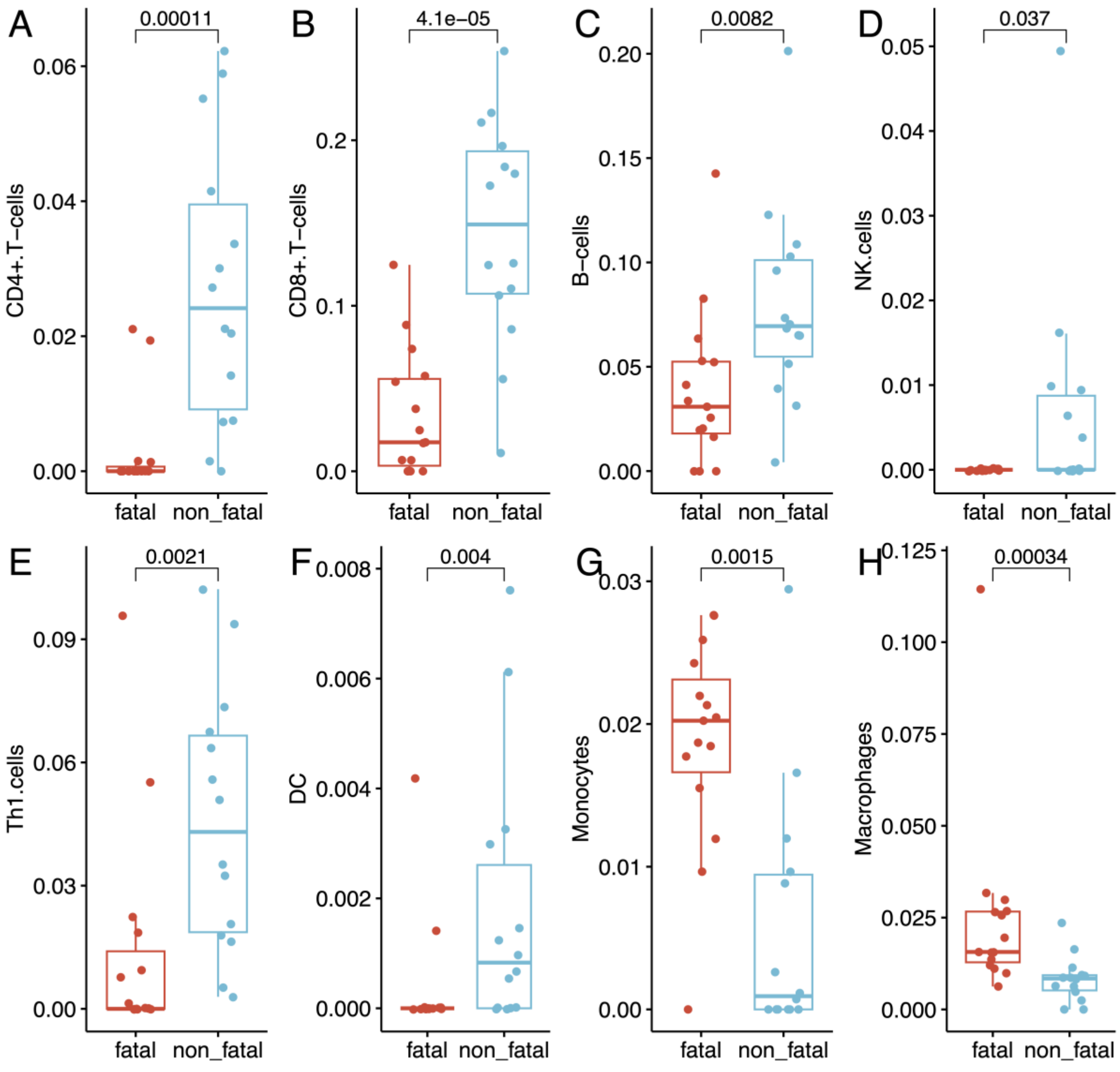

Supplemental Figure 9. Scattered boxplots show comparisons of imputed cell fractions between fatal (n=15) and non-fatal (n=14) melioidosis from the DORIM validation cohort. The Y-axis displays each cell type proportion. Non-parametric Mann-Whitney-Wilcoxon test was performed with its corresponding P-value displayed on each plot alongside median and inter-quartile range boxes.

Supplemental Table 1. Characteristics of Ubon-sepsis fatal and non-fatal melioidosis cases

Characteristic (N=164)	Fatal melioidosis (n=84)	Non-fatal melioidosis (n=80)	P value
Female, no. (%)	32 (38)	21 (26)	0.1
Age in years, median (IQR)	55 (45-67)	54 (46-64)	0.67
Diabetes, no. (%)	37 (44)	37 (46)	0.78
Bacteremia, no. (%)	76 (91)	54 (68)	<0.001
Pneumonia, no. (%)	33 (39)	25 (31)	0.28
Modified SOFA* score (IQR)	8 (5-11)	2 (1-5)	<0.001

*SOFA, sequential organ failure assessment; fatal and non-fatal cases were classified based on 28-day mortality

Supplemental Table 2. Top 25 predicted upstream regulators in fatal compared to non-fatal melioidosis patients in the Ubon-sepsis discovery cohort.

Fatal versus non-fatal melioidosis			
**Upstream Regulator**	**Predicted Activation State**	**Activation z-score**	**P-value of overlap**
TNF	Activated	3.627	4.31E-35
IL1B	Activated	4.383	3.26E-24
IL4	Activated	2.162	3.34E-23
CEBPA	Activated	3.199	1.84E-19
CSF3	Activated	2.134	6.55E-18
IFNG	Activated	2.663	1.04E-17
IL6	Activated	4.696	1.23E-17
IL1	Activated	3.24	4.23E-14
Tnf (family)	Activated	2.419	2.10E-13
EZH2	Activated	2.868	4.20E-13
IL1A	Activated	3.705	6.32E-13
PTGER2	Activated	2.14	6.13E-12
OSM	Activated	4.607	6.18E-12
TGFBR2	Inhibited	-2.489	1.05E-11
C5	Activated	2.527	3.98E-11
AGT	Activated	2.433	5.52E-11
NOD2	Activated	3.346	9.38E-11
IL17A	Activated	3.746	6.86E-10
IL33	Activated	2.142	1.32E-09
IKBKB	Activated	4.059	1.33E-09
CEBPB	Activated	2.243	1.45E-09
NFkB (complex)	Activated	3.331	1.49E-09
STAT1	Activated	2.226	1.58E-09
STAT3	Activated	2.046	1.89E-09
IL22	Activated	2.958	2.10E-09

Shared predicted upstream regulators between the two phenotypes are in bold. Only those upstream regulators considered significant when all criteria are met; satisfied predicted activation z-score (absolute value of 2), and P-value of overlap <0.01.

Supplemental Table 3. Characteristics of patients in the DORIM validation cohort.

**Characteristic (N=29)**	**Fatal melioidosis (n=15)**	**Non-fatal melioidosis (n=14)**
Female, no. (%)	4 (27)	4 (29)
Age in years, median (IQR)	55 (46-65)	50 (44-62)
Diabetes, no. (%)	8 (53)	7 (50)

This table was adapted from *Yimthin et al.* Emerging microbes & infections vol. 10,1 (2021): 8-18.

Supplemental Table 4. The top 20 canonical pathways in fatal compared to non-fatal melioidosis patients common to the Ubon-sepsis discovery cohort and DORIM validation cohort.

**Canonical Pathways**	**Discovery**	**Validation**
MSP-RON Signaling In Macrophages Pathway	3.742	1.387
PD-1, PD-L1 cancer immunotherapy pathway	3	2.558
IL-6 Signaling	2.138	2.683
Crosstalk between Dendritic Cells and Natural Killer Cells	-1.897	-3
CD28 Signaling in T Helper Cells	-2.449	-3
Glioblastoma Multiforme Signaling	-2.496	-1.886
GPCR-Mediated Nutrient Sensing in Enteroendocrine Cells	-2.5	-2
Basal Cell Carcinoma Signaling	-2.646	-1.633
Dendritic Cell Maturation	-2.746	-1.633
G-Protein Coupled Receptor Signaling	-2.769	-1.668
Factors Promoting Cardiogenesis in Vertebrates	-3.13	-2.183
Systemic Lupus Erythematosus In T Cell Signaling Pathway	-3.13	-2.646
Th1 Pathway	-3.13	-2.646
PKC(theta) Signaling in T Lymphocytes	-3.3	-2.6
Calcium-induced T Lymphocyte Apoptosis	-3.606	-4
ICOS-ICOSL Signaling in T Helper Cells	-3.606	-4.472
CREB Signaling in Neurons	-3.825	-3.263
Role of NFAT in Regulation of the Immune Response	-4.146	-3.157
Regulation of IL-2 Expression in Activated and Anergic T Lymphocytes	-9.916	-2.236
T Cell Receptor Signaling	-10.823	-4.217

Z-scores are shown with a cut-off absolute ≥ 2 to be considered significantly enriched based on IPA algorithm. Positive (orange) and negative (blue) z-scores indicate activation and inhibition of those pathways respectively.

Supplemental Table 5. Top 25 predicted upstream regulators in fatal compared to non-fatal melioidosis patients in the Ubon-sepsis discovery cohort and the DORIM validation cohort.

**Discovery cohort**			**Validation cohort**		
**Upstream Regulator**	**Activation z-score**	**p-value of overlap**	**Upstream Regulator**	**Activation z-score**	**p-value of overlap**
TNF	3.627	4.31E-35	***TNF***	2.484	1.79E-25
IL1B	4.383	3.26E-24	***IL4***	3.104	5.19E-23
IL4	2.162	3.34E-23	***IFNG***	2.276	1.58E-20
CEBPA	3.199	1.84E-19	***IL1B***	3.571	2.45E-20
CSF3	2.134	6.55E-18	Immunoglobulin	-4.484	1.16E-19
IFNG	2.663	1.04E-17	***CEBPA***	3.71	7.90E-18
IL6	4.696	1.23E-17	***CSF3***	4.662	4.21E-17
IL1	3.24	4.23E-14	***IL6***	4.202	1.03E-16
Tnf (family)	2.419	2.10E-13	*CSF2*	2.169	9.69E-16
EZH2	2.868	4.20E-13	TCR	-2.701	4.67E-15
IL1A	3.705	6.32E-13	*PGR*	3.007	2.29E-13
PTGER2	2.14	6.13E-12	***STAT3***	2.872	4.27E-13
OSM	4.607	6.18E-12	***OSM***	3.44	4.86E-13
TGFBR2	-2.489	1.05E-11	*CSF1*	2.109	8.22E-13
C5	2.527	3.98E-11	*SP1*	2.237	2.39E-12
AGT	2.433	5.52E-11	*KLF2*	-2.368	3.01E-12
NOD2	3.346	9.38E-11	***IL17A***	3.793	9.65E-11
IL17A	3.746	6.86E-10	*EGF*	4.093	2.49E-10
IL33	2.142	1.32E-09	*PPARG*	3.087	5.24E-10
IKBKB	4.059	1.33E-09	*TGM2*	5.549	5.62E-10
CEBPB	2.243	1.45E-09	Akt	2.614	7.91E-10
NFkB (complex)	3.331	1.49E-09	*MYD88*	2.045	2.16E-09
STAT1	2.226	1.58E-09	*NR3C1*	2.18	2.24E-09
STAT3	2.046	1.89E-09	*STAT6*	2.069	3.10E-09
IL22	2.958	2.10E-09	*HIF1A*	2.003	1.53E-08

Shared predicted upstream regulators between the cohorts are in bold. Only those upstream regulators considered significant when all criteria are met; satisfied predicted activation z-score (absolute value of 2), and P-value of overlap <0.01.

Supplemental Table 6. Sensitivity analysis of 5-gene panel performance for mortality prediction in the DORIM validation cohort compared to a clinical organ failure score.

	Model	AUC	95% CI
1	organ failure score	0.695	[0.480, 0.910]
2	organ failure score + diabetes + age + sex	0.652	[0.428, 0.877]
3	5-gene signature	0.833	[0.675, 0.991]

Supplemental References

1. T. Yimthin *et al.*, Blood transcriptomics to characterize key biological pathways and identify biomarkers for predicting mortality in melioidosis. *Emerg Microbes Infect* **10**, 8-18 (2021).
2. M. I. Love, W. Huber, S. Anders, Moderated estimation of fold change and dispersion for RNA-seq data with DESeq2. *Genome Biol* **15**, 550 (2014).
3. Y. Benjamini, Y. Hochberg, Controlling the false discovery rate: a practical and powerful approach to multiple testing. *Journal of the Royal statistical society: series B (Methodological)* **57**, 289-300 (1995).
4. Y. Liao, J. Wang, E. J. Jaehnig, Z. Shi, B. Zhang, WebGestalt 2019: gene set analysis toolkit with revamped UIs and APIs. *Nucleic Acids Res* **47**, W199-W205 (2019).
5. G. Yu, L. G. Wang, Y. Han, Q. Y. He, clusterProfiler: an R package for comparing biological themes among gene clusters. *OMICS* **16**, 284-287 (2012).
6. P. Langfelder, S. Horvath, WGCNA: an R package for weighted correlation network analysis. *BMC Bioinformatics* **9**, 559 (2008).
7. D. Aran, Z. Hu, A. J. Butte, xCell: digitally portraying the tissue cellular heterogeneity landscape. *Genome Biol* **18**, 220 (2017).
8. M. D. Robinson, D. J. McCarthy, G. K. Smyth, edgeR: a Bioconductor package for differential expression analysis of digital gene expression data. *Bioinformatics* **26**, 139-140 (2010).
9. T. Kaewarpai *et al.*, IL-1R2-based biomarker models predict melioidosis mortality independent of clinical data. *Front Med (Lausanne)* **10**, 1211265 (2023).
